# Successful management of recurrent erythema multiforme with upadacitinib: A case report

**DOI:** 10.1016/j.jdcr.2025.03.002

**Published:** 2025-03-17

**Authors:** Francelia Eckembrecher, Jayci G. Rhein, Sylvia Hsu

**Affiliations:** a University of Miami Miller School of Medicine, Holy Cross Health, Fort Lauderdale, Florida; b Department of Dermatology, Temple University Lewis Katz School of Medicine, Philadelphia, Pennsylvania

**Keywords:** recurrent erythema multiforme, upadacitinib

## Introduction

Recurrent erythema multiforme (EM) is a treatment challenge. We present a case of recurrent EM, refractory to numerous systemic agents, which cleared and remained clear upon initiation of upadacitinib.

EM is an uncommon acute, immune-mediated mucocutaneous condition that manifests as distinctive target-like lesions. EM is most commonly caused by herpes simplex virus (HSV) infection followed by *Mycoplasma pneumoniae* infection. The diagnosis of EM is clinical, as there is significant histopathologic overlap between this entity and other mucocutaneous eruptions, such as reactive infectious mucocutaneous eruption (RIME). EM, as well as RIME, may follow a recurrent or persistent course.

Clinically, EM presents as targetoid lesions with concentric color variation. It is usually distributed on the extensor surfaces of the extremities in a bilateral symmetric fashion.^1^^,^^2^ Mucosal involvement, typically in the oral mucosa, is common. Patients generally do not exhibit systemic symptoms.

## Case presentation

**Fig 1:**
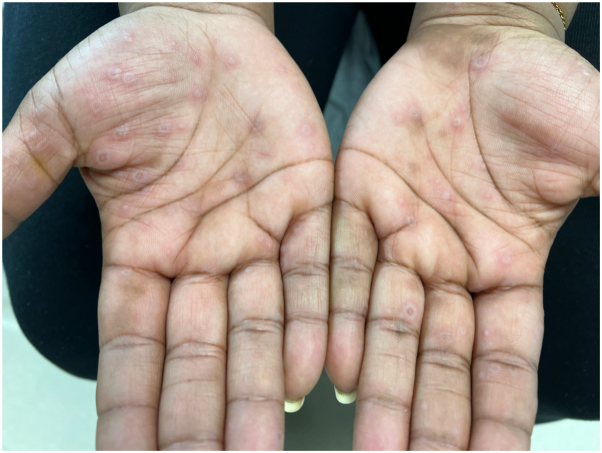
Targetoid papules on the palms.

**Fig 2:**
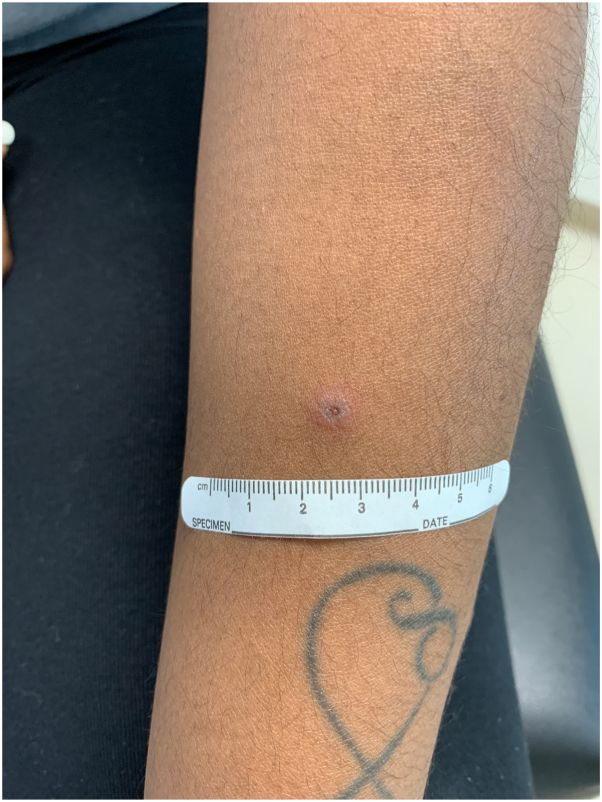
Dusky vesicular papule on the left dorsal forearm.

A 27-year-old African American woman without any past medical history presented with vesicular lesions on the bilateral palms. The lesions progressed to dusky erythematous papules and plaques on the palms (Fig 1) and soles, as well as erosions in the buccal mucosa and gingiva. She also had a single targetoid papule on her left forearm (Fig 2).

**Fig 3:**
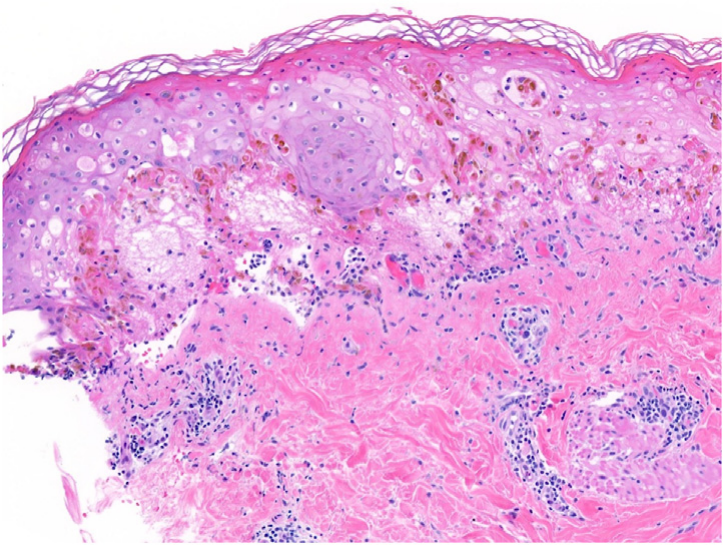
Histopathologic examination of the lesion on the left forearm, showing a vacuolar interface dermatitis with superficial perivascular lymphocytic infiltrates and dyskeratosis of keratinocytes, consistent with erythema multiforme (hematoxylin and eosin 100×).

Two 4-mm punch biopsies, one of her palm and the other one of her forearm, showed a vacuolar interface dermatitis with superficial perivascular lymphocytic infiltrates and dyskeratosis of keratinocytes, consistent with EM (Fig 3).

Over a span of 3 years, the patient had 20 flares of EM. Laboratory evaluations showed positive HSV2 IgG and Mycoplasma IgG, while Mycoplasma IgM and *Mycoplasma pneumoniae* polymerase chain reaction were negative. As she lacked upper respiratory symptoms, she was not treated with antibiotics. As our patient had a classic presentation of EM and did not have 2 or more mucosa involved, mycoplasma-induced rash and mucositis/RIME were not favored.

She was started on prophylactic valacyclovir, initially on 500 mg daily, later increased to 1 g daily, but without improvement. She was treated with many medications over the next 2 years. Clobetasol 0.05% ointment was applied to the hands without any improvement. Prednisone up to 60 mg/day cleared her eruption. However, when she discontinued her prednisone, her EM would recur. She was then started on cyclosporine 300 mg, which she took for 3 months without any clinical improvement. Dapsone 100 mg was trialed for 2 months but did not provide any improvement. Subsequently, she was placed on mycophenolate mofetil 1 g twice a day for 8 months without any improvement. The patient had numerous courses of prednisone of 40 to 60 mg/day with complete clearance followed by rapid recurrence after tapering off the drug. Given her recurrences, she was started on upadacitinib 15 mg orally daily, which led to complete clearance within a week. The patient's eruption recurred within 3 days when she ran out of upadacitinib; otherwise, she has remained clear. She has been taking upadacitinib for over a year. She wants to continue taking upadacitinib because her eruption recurred when she ran out of the drug.

## Discussion

The mechanism of EM pathogenesis has been described in HSV-induced EM. It is believed to result from a cell-mediated immune reaction against viral antigen-positive cells containing the HSV DNA polymerase gene.^2^

Recurrent EM is defined as 6 or more episodes per year, with 61% to 100% of cases caused by HSV infection. A Mayo Clinic study found that 60% of cases were idiopathic, but some patients may have had subclinical HSV infections.^2^

The diagnosis of EM is made clinically, as there is significant histopathologic overlap with other mucocutaneous eruptions, notably *Mycoplasma pneumoniae*-induced mucocutaneous eruption/RIME.

RIME typically affects younger patients (mean age 11.9 years) and is predominantly a mucosal eruption. Proposed diagnostic criteria require the involvement of 2 or more mucosal sites to make the diagnosis. When the skin is involved, the body surface area that is affected is minimal.

The diagnosis of EM is based on the distribution of skin lesions, which are classically bilateral and symmetric and affect the extremities, including the palms, dorsal hands, elbows, knees, and feet. Mucosal involvement may be present but does not typically predominate.

Recurrent EM poses a significant treatment challenge. Antiviral prophylaxis is the primary treatment. In a study of 20 patients with EM, 7 of 11 in the daily acyclovir arm did not experience recurrence while taking the drug.^3^ Recurrence is frequently observed if antivirals are stopped.^4^

Prophylactic antiviral treatments include valacyclovir (500 mg daily or twice a day), famciclovir (250 mg twice a day), or acyclovir (400 mg twice a day). Responsive patients are advised to continue treatment for 1 to 2 years. Should EM recur after discontinuation, treatment should resume for an additional 6 to 12 months. Dosages may be doubled for nonresponsive patients.

Alternative options for those resistant to antiviral treatments include azathioprine, dapsone, mycophenolate mofetil, immunoglobulin, hydroxychloroquine, thalidomide, and cyclosporine.^2^

Our patient was treated with upadacitinib, a Janus kinase (JAK) inhibitor. It affects the JAK-STAT pathway by binding to JAK1, blocking the phosphorylation and activation of STAT, thereby disrupting the proinflammatory cytokine signaling cascade.^5^ Upadacitinib is used for various conditions in dermatology, gastroenterology, and rheumatology, including atopic dermatitis, psoriatic arthritis, nonradiographic axial spondyloarthritis, ankylosing spondylitis, rheumatoid arthritis, Crohn disease, and ulcerative colitis.^6^

## Conclusion

We present a case of a patient suffering from recurrent EM who had tried numerous medications without improvement. She cleared with the initiation of upadacitinib. Successful treatment of this patient with recurrent EM with a JAK inhibitor supports previous reports of this therapeutic option for recalcitrant disease.^7^^,^^8^

## Conflicts of interest

None disclosed.
